# Papillary Carcinoma of Breast: Clinicopathological Characteristics, Management, and Survival

**DOI:** 10.1155/2022/5427837

**Published:** 2022-10-13

**Authors:** Bushra Rehman, Anam Mumtaz, Barka Sajjad, Namra Urooj, Sameen Mohtasham Khan, M. Toqeer Zahid, Huma Mannan, M. Zulqarnain Chaudhary, Amina Khan, M. Asad Parvaiz

**Affiliations:** ^1^SKMCH & RC, Department of Breast Surgery, Lahore, Pakistan; ^2^SKMCH & RC, Department of Surgical Oncology, Lahore, Pakistan

## Abstract

**Objective:**

To study clinicopathological features, treatment strategies, and prognosis of papillary carcinoma of breast. *Material and Methods.* Data from 58 patients were retrospectively reviewed from January 2010 to December 2016. Four types of papillary carcinoma (on final resected specimen) were included, i.e., invasive papillary carcinoma (IPC), intracystic (encapsulated) papillary carcinoma (EPC), solid papillary carcinoma (SPC), and papillary DCIS (ductal carcinoma in situ). Various features of the four types were observed and compared.

**Results:**

Of the 58 patients, 8 were males (13.7%). The mean age at presentation was 61 years; the mean tumor size was 33 mm. The frequency of each histological type was as follows: IPC (*n* = 22/38%), EPC (*n* = 22/38%), SPC (*n* = 12/20.6%), and papillary DCIS (*n* = 2/3.4%). Only two patients were ER negative (both IPC). HER-2 Neu was positive in 3 patients only, out of which 2 died of progressive disease (one EPC and one IPC). LN metastasis was present in 3 (5%) patients (one in each of 1st three types) and only one died of bone metastasis that was also Her-2Neu positive. All patients underwent upfront surgery except two patients who had synchronous IDC on the contralateral side. Breast conservation surgery (BCS) was performed in 34 (58.6%) and mastectomy in 22 (37.9%) patients. 13 patients did not undergo invasive axillary staging; the rest of 43 (74%) patients did (32 sentinel biopsy and 11 axillary dissection). Chemotherapy was given to 18 patients (31%), mostly to IPC (*n* = 12). Only 2 patients had bone metastasis (one was IPC and one EPC). Cancer-related death was observed in 3 patients. For all groups combined, 5-year OS was 98% and DFS was 92%.

**Conclusion:**

Overall, papillary carcinoma of the breast has an excellent prognosis, even though less intense treatment modalities were used. It is still difficult to define the optimum management and avoid overtreatment, given the limited data in the literature.

## 1. Introduction

Breast and lung cancers are the two most common cancers worldwide (contributing 12.5% and 12.2% of the total number of new cases diagnosed in 2020) [1]. The incidence of breast cancer is 1 in 9 women at some stage of their life [2].

Papillary carcinoma of the breast is an exceedingly rare subtype of breast cancer, representing approximately 1% of cases of carcinoma breast [3, 4].

Under the microscope, the defining morphological feature of these lesions is the presence of arborescent fibrovascular cores/fronds/papillae. A layer of intervening myoepithelial cells in the fibrovascular fronds distinguishes benign (papilloma) from malignant (absent in malignant, i.e., negative P63 marker) [5].

There is variable appearance of papillary carcinoma on various imaging modalities, and this makes the differentiation of benign from malignant pathologies difficult on imaging, and tissue sampling is warranted [6]. However, on core biopsy, papillary carcinoma is usually categorized as B3 lesions (uncertain malignant potential), and complete excision is necessary to establish accurate diagnosis [7].

The 5th edition of WHO 2019 classifies papillary neoplasms into 7 subtypes [8], in which the 1st one is benign (excluded from this study). The rest of the 6 types fall under the category of *papillary carcinoma*. Types 2 to 6 (papillary DCIS, EPC, and SPC) are considered equivalent to ductal carcinoma in situ (DCIS) without or with invasion, while 7th type which the invasive papillary carcinoma (IPC) is considered an invasive cancer.

1. Intraductal papilloma, 8503/0
2. Ductal carcinoma in situ, papillary, 8503/2
3. Encapsulated papillary carcinoma, 8504/2
4. Encapsulated papillary carcinoma with invasion, 8504/3
5. Solid papillary carcinoma in situ, 8509/2
6. Solid papillary carcinoma with invasion, 8509/3
7. Intraductal papillary adenocarcinoma with invasion, 8503/3

Clinical behavior and treatment-related information for patients with papillary carcinoma is limited. There are no guidelines or widely agreed consensus on its management protocols. The scarcity of information necessitates the need for further treatment- and outcome-related studies in papillary carcinoma of the breast.

The study is aimed at contributing to the already sparse information due to its rare occurrence and at better understanding its behavior.

## 2. Methodology

During 1 January 2010 to 31 December 2016, all patients with papillary carcinoma presented at Shaukat Khanum Memorial Cancer Hospital and Research Center (SKMCH&RC) were selected. This was a retrospective study with convenience sampling. Ethical approval was sought from the Institutional Review Board (IRB) of SKMCH&RC.

The case selection was done based on final histology after curative surgery, all types of papillary neoplasms (except intraductal papilloma) were included, there was no exclusion criterion, and distant metastasis cases were also included.

Various features of the four types were observed and compared. These included age, gender, receptors, tumor size, nodal status, and mode of treatment given (type of surgery, chemotherapy, and hormonal therapy).

Tumor size was recorded from ultrasound performed at presentation. Nodal status was assessed on ultrasound and on FNAC (fine needle aspiration cytology) if indicated. Surgery types included mastectomy or modified radical mastectomy (MRM) and breast conservation surgery (BCS). In both cases, simultaneous axillary staging surgery was done (either axillary dissection or sentinel lymph node biopsy (SLNB)).

Most cases were diagnosed on core needle biopsy (CNB) as papillary neoplasm, which is designated as B3 lesion (uncertain malignant potential) according to the B-coding system, which means they should undergo complete excision for definite diagnosis, while some cases were diagnosed on incisional/excisional biopsy, having definitive diagnosis at the start.

The patients were followed up for a minimum of 5 years to a maximum of 10 years in some cases. Patterns of local recurrence, nodal, and distant metastasis were noted.

Data were collected through the human information system (HIS), an electronic database of the hospital. As the patient information are collected in real time and stored, it allows for the accurate retrospective review of the data.

Data analysis was performed using the Statistical Package for Social Sciences (Version 26.0). The survival functions were calculated by using the Kaplan-Meier curve.

## 3. Results

A total of 58 patients were included in the study, between 2010 and 2016. Four types were included, as given in Table 1.

The mean age at the presentation was 61 years (youngest 29 years and oldest 98 years). The mean size at the presentation was 33 mm. Distinctive characteristics of tumor are shown in detail in Table 2.

Out of 58 patients, eight were male, and all showed an excellent prognosis at 5 years. All patients were ER positive, except two patients who were ER negative (and PR negative). In two patients, ER status was not known, one was given tamoxifen empirically, and one was not given. ER positivity was strong in the majority; only 10 of 58 patients were less than 80% positive. The rest were more than 80% positive.

PR was negative in four patients only.

Both ER-negative patients were young females below 35 years. Both had IPC, underwent BCT/adjuvant chemo/XRT, and remained well.

HER-2 Neu testing was performed only in 37 patients (63.7%); only three patients were H2N positive. One was having IPC, and two were EPC. None received Herceptin. Two of them were elderly females who presented with advanced disease (one with T4/fungating mass (IPC) and one with N2/ulcerated nodal mass (EPC)) and only received hormonal treatment, and both died at 3 to 5 years with bone metastasis and progressive disease. The third patient was 55 years old and did well (who had breast conservation surgery/adjuvant systemic/XRT/tamoxifen).

Most patients presented with breast lump, only 10 patients had nipple discharge as the main complaint, and two were detected on screening.

The commonest T stage was T1 and T2, i.e., 5 cm or less. However, bigger masses and skin changes (ulceration) are also there as shown in Table 2.

Lymph node metastasis was present in three patients, each one of three main types. Only one patient who was 80 years old with EPC died of progressive disease and bone metastasis, also discussed above. She was Her-2Neu positive/ER/PR positive, T1N2M0 with a large axillary ulcerated mass, and was put on hormonal therapy only (no systemic).

As shown in Table 3, prognosis was good in terms of 5-year OS and DFS. Local recurrences were observed within 1 year of treatment in two patients only. One underwent reexcision (as XRT was not given initially), and one underwent mastectomy. Both remained well after 5 years. Bony metastasis was present in 2 cases; both died at 5 years.

As shown in Table 4, in 32 patients, SLNB was performed (only in one case, it was positive and followed by ALND (0/17LNs on ALND)). 13 patients did not undergo invasive axillary staging and was negative on ultrasound and mammogram. 11 patients underwent ALND (as part of MRM). 22 patients underwent mastectomy in which 8 were male patients. The rest underwent BCS (+/− axilla surgery).

*Chemotherapy* was administered to 12 patients with invasive papillary carcinoma (out of 21 IPC). Among the rest of the types, there were some indications of chemotherapy in those 6 cases (either element of invasion or contralateral IDC).

*XRT* (*radiation therapy*) was given to 3 mastectomy patients, all with invasive papillary carcinoma (2 were male patients-to chest wall; 1 female with positive axilla). Four BCS patients did not receive XRT, 2 refused, and 2 were not offered due to age > 80.

*Hormonal therapy in the form of Aromatase Inhibitor was given to 16 patients, rest received Tamoxifen*.

Various characteristics in 4 histological types were compared as shown in Table 5. Most of the patients were older, i.e., > 60 years; particularly, EPC and SPC were more common at >70 years. Males showed more IPC (6 out 8). ER/PR negativity was rare, 2 patients only, both IPC. Although SPC and EPC are slow growing, few of them were presented at T3 and T4 stages. LN positivity was shown, one in each of the three main types. Chemotherapy was not frequently used; only 30% of patients received it, mostly IPC.

Mastectomy (especially MRM) seems more aggressive for this favorable tumor type; however, 9 patients were with tumor size > 5 cm (T3 and T4), 8 were male patients, one was with local recurrence after undergoing breast conservation and radiation, one had multiple margins positive on excision, and one was due to patient wish (for B/L mastectomy), while both patients of papillary DCIS had extensive calcifications. In all these (*n* = 22), upfront mastectomy was a better option. Radiation and hormonal treatment were administered to BCS and all ER-positive patients irrespective of histological type.

## 4. Discussion

Papillary carcinoma is considered a low-grade carcinoma. Given the rarity of the disease and confusion regarding its histological classification, there is no widely accepted consensus on the management of patients with papillary carcinoma. This study studies the clinic-pathological characteristics as well as outcomes and treatment strategies used for patients with papillary carcinoma in our set-up.

Usually, all these lesions are diagnosed as papillary lesions on core biopsy, which falls in the category of B3 lesions necessitating surgical excision for accurate diagnosis [8]. In this study, most of the patients had papillary lesions on core biopsy; however, few had incisional/excisional biopsy at presentation.

In male gender, papillary carcinoma is relatively more common. In our study, male ratio was higher (13.7%), compared to other studies in which 3.5% cases were male [9, 10].

Papillary breast cancer typically presents with a bloody nipple discharge, an abnormal mass, or radiographic abnormalities [8, 10]. In our study, 17% presented with a bloody nipple discharge, while the rest came with a breast lump.

The median age of patients in this study was 61 years (youngest 29 years and oldest 98 years). Several previous studies have similarly shown that it frequently occurs in elderly postmenopausal women [8, 10].

They are usually strong receptor positive [11]. In this study, only 2 patients (3%) were ER negative, both were invasive papillary, both were young females, and none of the encysted or solid variety was ER negative. The HER 2Neu expression/positivity is also rare; in one study, none of the 39 patients with EPC had HER 2Neu expression [12]. In this study, however, 3 patients were HER 2Neu positive, one was IPC, and two were EPC.

LN metastasis does not occur frequently; in one study, it was 5.5% in EPC [11]; in our study, it was 4.5% in EPCs (one patient out of 12); and overall, for all types, it was 5.1% (3 of 58 patients).

In this study, 22 patients underwent mastectomy (axilla staged by SLNB in 11 and by axillary clearance in 11). They were all those who were accepted in the hospital system after their 1st diagnosis by incisional or mostly by excisional biopsy; otherwise, on core needle biopsy, decision of mastectomy cannot be made. The mastectomy we believe was an overtreatment, but there were some indications, as mentioned in Results. While axillary clearance in those 11 patients (only one followed positive SLNB) was not justified, as axilla was negative in 10 patients at final histopathology, these patients presented around 2010, when the department was evolving and management of papillary carcinoma was less defined.

Regardless of papillary carcinoma occurring in its pure form, or accompanied by DCIS or invasion, the prognosis remains excellent with almost nonexistent lymphovascular invasion [8] and a low rate of axillary and distant metastases [11]. In our study, the 5-year overall survival for all types together was 98%, while 6.5-year survival was 94%.

The three mortalities observed were elderly females who did not receive any systemic treatment, were all receptor positive, and received hormonal treatment. Two of them were IPCs and one was EPC. Poor prognostic factors were H2N positivity and LN positivity in one. Two of them had bone metastases. Now, we will discuss clinic-pathological features and treatment strategies of each type separately.

Papillary DCIS lacks an intact myoepithelial cell layer in papillae unlike intraductal papilloma. The myoepithelial cell layer is retained at the periphery of the involved duct wall, thus defining the in situ nature of the lesion (unlike EPC and SPC in which it is not intact). In this study, only 2 patients had papillary DCIS; both were in their 60s and were receptor positive. Both underwent mastectomy (due to widespread calcification), received adjuvant hormonal therapy, and remained well.

Encapsulated papillary carcinoma (EPC) is also known as intracystic papillary carcinoma; it arises in a cystically dilated duct. It can be with invasion or without invasion (as shown in WHO classification, nos. 3 and 4). EPC without invasion is considered a variant of DCIS; however, a myoepithelial cell layer at the periphery (a feature of DCIS) may not be consistently demonstrated on immunohistochemistry [8], but they still have an indolent behavior even with invasion [11]. EPC invasion involves infiltration beyond the fibrous capsule and an associated stromal reaction. In our study, 22 patients (38%) had EPC, out of which only 3 patients had an element of invasion. Grabowski et al. established the results of a large series of 913 patients emphasizing that long-term survival was good irrespective of invasion [13]. As shown in Table 5, ER was positive in all, while Her-2Neu was positive in 2 patients, and one had LN metastasis. This shows that Her-2Neu testing should be done along with ER/PR; axilla, although rarely involved, should be staged. Hormonal therapy was given to all, while adjuvant systemic therapy was given to 3 patients only, those having invasion. Radiation therapy was given as part of BCS that was performed in 17 patients. Although few researchers believe, surgical excision with clear margin is all that needed in pure EPC without invasion omitting hormonal or radiation therapy, especially in older age [11, 12]. In another study, Mogal et al. found that radiation therapy improved disease-free survival [14]. In our set-up, we followed a more conventional method of treatment that included staging of the axilla and adequate excision followed by radiation and hormonal therapy. In our study, one patient had local recurrence (no radiation initially), and one had bony metastasis and subsequent mortality. Summing up, EPC has a good prognosis even if elements of invasion are present. Surgical excision with adequate margin is the mainstay of treatment with axillary staging preferably in younger patients. It should be followed by radiation and hormonal therapy, especially in younger patients. The routine use of systemic therapy is not appropriate.

Solid papillary carcinoma (SPC) is circumscribed, densely cellular, and expansile nodule of epithelial cells (WHO classification nos. 5 and 6). Although no papillary structures are present, an underlying fibrovascular stromal network is typically observed, supporting the classification of the lesion as papillary, despite its solid morphological appearance. Like EPC, solid papillary carcinoma without invasion is considered a variant of DCIS; however, a myoepithelial cell layer at the periphery may be lacking in some [15]. SPC with invasion frequently manifests as a mucinous or neuroendocrine-like carcinoma; the presence of invasion has slightly more incidence of metastasis; however, overall, it still has an indolent course [16, 17]. Like EPC, it is also more common in elderly females, almost always ER positive and a very low incidence of LN metastasis (3%) [17]. In our study, 12 patients had SPC, all in elderly females (except one who was 40 years old); all were ER positive, and none was Her-2Neu positive. One patient had LN metastasis, and 3 patients received systemic therapy due to the presence of invasion. All patients received hormonal treatment and radiation as part of BCS (9 patients). None of the patients had distant metastasis or mortality. Summing up, SPC should be managed with surgical excision with negative margins, followed by radiation and hormonal therapy. Systemic therapy can be avoided.

The clinic-pathological data is even more scarce in invasive papillary carcinoma (IPC) (WHO classification no. 7), although it is a more common subtype. Much of the published literature about IPC in fact describes variants of encapsulated or solid papillary carcinoma with invasion [18, 19]. IPC is considered a variant of invasive ductal carcinoma with infiltrative papillary growth, but it should not be confused with invasive micropapillary carcinoma that lacks true fibrovascular cores and is an aggressive form of mammary carcinoma.

In our study, out of 22 patients with IPC, majority of women were old age, but 2 patients were younger than 40 years. Male patients were 6. Two patients were ER and PR negative, while one was Her-2Neu positive. Advanced T stage (T3/T4) was in 4 patients; only one patient was LN positive. Chemotherapy in an adjuvant setting was administered to 12 patients (54%). Two patients died of progressive disease. Summing up, IPC has a better prognosis than invasive ductal carcinoma; however, systemic therapy should still be offered.

After reviewing the literature and our own patient experience, we concluded the following:

ER/PR receptor status should be known in all cases, although it is almost always receptor positive, but rarely, is it not (2 cases in our study, both IPC). Similarly, Her-2Neu status should be known too, as few cases can be positive (3 cases in our study, one IPC and two EPC); however, Herceptin role is uncertain (not given to any in our patients). LN involvement is also a rare phenomenon (in our study, only 3 were LN positive); axillary staging by surgery should be performed especially in younger females; in our study, axillary staging was done in 43 patients (74%).

Papillary DCIS should be treated like any DCIS. Intracystic papillary carcinoma or solid papillary carcinoma should not be treated on aggressive lines of invasive mammary carcinoma; even with an invasion, the prognosis remains good. Surgical excision with a clear margin is the mainstay of treatment for both. Axillary staging, radiation as part of breast conservation, and hormonal treatment should be given in younger women.

However, invasive papillary carcinoma is treated on conventional lines as a mammary carcinoma; however, in older patients, systemic therapy can be avoided. In our study, almost half of the patients (54%) with IPC received adjuvant systemic therapy (which were less than 65 years of age).

Concluding the discussion, papillary carcinoma has better prognosis, and treatment modalities should also be less aggressive as compared to invasive mammary carcinoma and should be individualized based on the age of the patient.

## Figures and Tables

**Table 1:** Types of papillary carcinoma in study.

	Type	Numbers (total = 58)
1.	Invasive papillary carcinoma (IPC)	A total of 22 (38%)
2.	Intracystic/encapsulated papillary carcinoma (EPC)	A total of 22 (38%)
3.	Solid papillary carcinoma (SPC)	A total of 12 (20.6%)
4.	Papillary DCIS	A total of 2 (3.4%)

**Table 2:** Distinctive/unusual features observed.

Characteristics	Numbers	Subtypes	Outcomes
Gender			

Male	A total of 8 (14%)	Among 8 males, six ➔ IPC and two ➔ EPC	All 8 did well (all mastectomies)
Female	A total of 50 (86%)		

ER negative	A total of 2	Both were young females < 35 Both IPC	Did well

PR negative	4 patients		

H2N positive (37 patients had H2N testing)	A total of 3	One ➔IPC Two ➔ EPC	Two died due to distant metastasis (one IPC and one EPC)

T stage at presentation (T3 and T4)	T1 (*n* = 15) T2 (*n* = 34) T3 (*n* = 7) T4 (*n* = 2)	SPC and EPC were also there in T3 and T4	No mortality in T1 Much mortality in rest of T stages

LN status at presentation Only 3 patients were LN positive	One each of SPC, IPC, and EPC	(1) SPC (FNA positive/T4) (2) IPC (SLNB positive/T2)	Only EPC (3^rd^) died at 5 years of bone
		(3) EPC (fixed ulcerated axillary mass/FNA positive/T2/also Her-2Neu positive)	Metastases, rest of two remained well.

**Table 3:** Recurrences and survival in four types.

Prognosis	Numbers	Subtypes	Outcomes
Local recurrences	A total of 2	IPC EPC	Both remained well

Distant metastasis (both bony)	A total of 2	IPC EPC	Both died

Mortality	A total of 6 (3 primary cancer-related deaths)	2 died of bone metastasis 2 died of renal failure, 1 died of renal mass of uncertain origin, and 1 died of uncertain reason Initially, fungating mass did not complete treatment	So primary cancer-related deaths were in 3 patients (IPC × 2 (EPC × 1)

Overall survival (OS)	98%		

Disease-free survival (DFS)	92%		

**Table 4:** Different treatment modalities.

Type of treatment	Number of patients	Subtypes of treatment	
Surgery	Surgery was done in all except in two (both were locally advanced)	Mastectomy+SLNB (9) Simple mastectomy (2) MRM (11) Reexcision+SLNB (10) WLE+SLNB (13) WLE only (11)	SLNB = 32 patients ALND = 11 patients (as part of MRM) Axillary staging by surgery 43 patients (74%)

Chemotherapy	Yes = 18 patients (31%) No = 40 patients (69%)	IPC = 12 EPC = 3 SPC = 3	Neoadjuvant = 2 patients only (both had contralateral IDC)

Radiation (XRT)	A total of 33 patients (57%) received XRT	All BCS & 3 Mastectomy patients	4 BCSs did not receive XRT due to age > 80

Hormonal treatment	All patients except (1) two ER/PR negative and (2) one with unknown hormonal status	Aromatase inhibitor = 16 patients Tamoxifen = 39 patients	

Abbreviations: MRM: modified radical mastectomy; WLE: wide local excision; ALND: axillary lymph node dissection; SLNB: sentinel lymph node biopsy; XRT: radiation therapy.

**Table 5:** Various characteristics among 4 tumor types compared.

	IPC (*n* = 22)	EPC (*n* = 22)	SPC (*n* = 12)	Pap DCIS (*n* = 2)
Age				
<40	2	2	1	0
40–70	16	13	8	2
>70	4	7	3	0
Male gender (*n* = 8)	6	2	0	0
ER negative (*n* = 2)	2	0	0	0
PR negative (*n* = 5)	2	2	1	0
Her-2Neu (*n* = 3)	1	2	0	0
Advanced T stage				
T3 (*n* = 7)	3	3	1	0
T4 (*n* = 2)	1	1	0	0
LN positive (*n* = 3)	1	1	1	0
Chemotherapy (*n* = 18)	12	3	3	0
Surgery				
Mastectomy (*n* = 22)	14	4	2	2
BCS (*n* = 34)	8	17	9	0
XRT (*n* = 33)	11	15	7	0
Mortality	2	1	0	0
	1➔ Her-2Neu positive and T4 1➔ bone metastasis	Her-2Neu/LN positive/bony metastases		

Abbreviations: BCS: breast conservation surgery; LN: lymph node.

## Data Availability

The [SPSS] data used to support the findings of this study are included within the article.
